# Effect of Vitamin C and Protein Supplementation on Plasma Nitrate and Nitrite Response following Consumption of Beetroot Juice

**DOI:** 10.3390/nu14091880

**Published:** 2022-04-29

**Authors:** Gary D. Miller, Beverly A. Nesbit, Daniel B. Kim-Shapiro, Swati Basu, Michael J. Berry

**Affiliations:** 1 Department of Health and Exercise Science, Wake Forest University, Winston-Salem, NC 27109, USA; berry@wfu.edu; 2 Translational Science Center, Wake Forest University, Winston-Salem, NC 27109, USA; nesbitba@wrfu.edu (B.A.N.); shapiro@wfu.edu (D.B.K.-S.); basus@wfu.edu (S.B.); 3 Department of Physics, Wake Forest University, Winston-Salem, NC 27109, USA

**Keywords:** nitrate, nitrite, nitric oxide, beetroot juice, vitamin C

## Abstract

Beetroot juice is a food high in nitrate and is associated with cardiometabolic health benefits and enhanced exercise performance through the production of nitric oxide in the nitrate–nitrite–nitric oxide pathway. Since various food components influence this pathway, the aim of this trial was to study the effect of beetroot juice alone and in conjunction with vitamin C or protein on the acute response to plasma nitrate and nitrite levels in healthy middle- to older-aged adults. In this cross-over trial, each participant received, in a randomized order, a single dose of Beet It Sport^®^ alone; Beet It Sport^®^, plus a 200 mg vitamin C supplement; and Beet It Sport^®^ plus 15 g of whey protein. Plasma levels of nitrate and nitrite were determined prior to and at 1 and 3 h after intervention. Log plasma nitrate and nitrite was calculated to obtain data that were normally distributed, and these data were analyzed using two-way within-factors ANOVA, with time and treatment as the independent factors. There were no statistically significant differences for log plasma nitrate (*p* = 0.308) or log plasma nitrite (*p* = 0.391) values across treatments. Log plasma nitrate increased significantly from pre-consumption levels after 1 h (*p* < 0.001) and 3 h (*p* < 0.001), but plasma nitrate was lower at 3 h than 1 h (*p* < 0.001). Log plasma nitrite increased from pre to 1 h (*p* < 0.001) and 3 h (*p* < 0.001) with log values at 3 h higher than at 1 h (*p* = 0.003). In this cohort, we observed no differences in log plasma nitrate and nitrite at 1 h and 3 h after co-ingesting beetroot juice with vitamin C or a whey protein supplement compared to beetroot juice alone. Further research needs to be undertaken to expand the blood-sampling time-frame and to examine factors that may influence the kinetics of the plasma nitrate to nitrite efficacy, such as differences in fluid volume and osmolarity between treatments employed.

## 1. Introduction

Inorganic nitrogen compounds, principally nitrate, are found in beetroot and many leafy green vegetables, such as spinach and lettuce [1]. Beetroot juice is a food high in nitrate and is associated with cardiometabolic health benefits and enhanced exercise performance through the production of nitric oxide in the nitrate–nitrite–nitric oxide pathway [2,3,4]. The physiological effects of nitrate are attributed to its endogenous nitric oxide formation, a molecule with important vascular and metabolic functions [5,6,7]. These actions include regulation of blood flow, muscle contractility, inhibition of platelet aggregation, prevention of adhesion of inflammatory signals and cells, improving endothelial barrier cell function, muscle glucose uptake, angiogenesis, decreased level of oxygen cost during exercise, and enhanced exercise performance [2,8,9,10,11,12,13], and are attributed to both cyclic guanosine monophosphate (cGMP)- dependent and cGMP-independent effects of nitric oxide [14,15,16,17,18,19]. Nitric oxide is formed through two pathways in the body. The first is through the oxidative reactions of nitric oxide synthase and arginine to form nitric oxide and citrulline [20]. The second method is through the nitrate–nitrite–nitric oxide pathway [21]. In this latter pathway, nitrate ingested from food is nearly 100% absorbed in the small intestine to the portal vein [7,22,23]. Twenty-five percent of this absorbed nitrate is then actively transported into the salivary glands, where the nitrate is reduced to nitrite by anerobic facultative bacteria located on the posterior aspect of the tongue [24,25]. The salivary nitrite is swallowed and absorbed into the circulation [7,26,27,28,29]. Acute and chronic intake of nitrate in its various forms, including beetroot juice, green leafy vegetables, or as a nitrate salt, substantially raises nitric oxide metabolites, such as plasma nitrate and nitrite levels [7,30], with the peak plasma response occurring from 1–3 h after ingestion of the nitrate source [31,32,33].

Endothelial dysfunction, manifested as a reduction in nitric oxide production via nitric oxide synthase pathway, is apparent with aging, primarily through prominent increases in inflammation and oxidative stress [34,35,36]. More specifically, older adults have lower levels of both L-arginine, the nitric oxide synthase substrate, and tetrahydrobiopterin, a cofactor for the reaction [37,38]. Additionally, aging is positively associated with increases in asymmetric dimethylarginine, an endogenous inhibitor of nitric oxide synthase [39]. Thus, optimizing nitric oxide generation through the alternative nitrate–nitrite–nitric oxide pathway is a reasonable goal to improve cardiovascular health [40,41,42].

Additionally, plasma nitrate and nitrite responses are known to be influenced by the consumption of components of foods, including polyphenols, antioxidants, and fatty acids [43,44,45]. Vitamin C has long been known to interact with the chemical reactions of nitrite [44]. Briefly, nitrite in an acidic environment, such as the stomach, can form nitrous acid (HNO_2_), which can be converted to nitrosamides (HN_2_O_2_) and nitrosamines (H_2_N_2_O) through dinitrogen trioxide (N_2_O_3_) as an intermediate [33,46]. Both nitrosamides and nitrosamines are carcinogenic. The presence of vitamin C in the stomach reduces the conversion of nitrite to N-nitroso compounds by reducing the nitrite to nitric oxide, which diffuses out of the gastric tract, influences gastric blood flow, and is reconverted to nitrite. Both protein and beetroot juice are frequently consumed supplements of athletes to enhance exercise performance. Since they may be consumed simultaneously, it is important and logical to assess if there is an interaction that would either potentiate or negate dietary nitrate’s effect on plasma nitrite. In an acidic environment of the stomach, the amine group in the amino acids of protein react with N_2_O_3_ to form nitrosamines [33,46]. This would potentially reduce plasma nitrite levels, as less nitrite would be available for absorption. Currently, research does not indicate that dietary protein affects the metabolism of nitrate in the nitrate–nitrite–nitric oxide pathway, such as reducing or increasing plasma levels of nitrate or nitrite. Thus, the aim of this trial was to study the effect of beetroot juice alone and in conjunction with vitamin C or protein on the acute response to plasma nitrate and nitrite levels in middle- to older-aged adults. It was hypothesized that compared to beetroot juice alone, the co-ingestion of vitamin C and beetroot juice would lead to higher plasma nitrite levels, and consuming protein along with beetroot juice would lead to lower plasma nitrite levels.

## 2. Methods

### 2.1. Participant Selection

Healthy adults (body mass index 18.5–30.0 kg/m^2^) 40–80 years of age were recruited from the Winston-Salem, North Carolina Community. A recruitment flyer was placed around the Wake Forest University campus and distributed to the Winston Salem community. Inclusion and exclusion criteria are presented in Table 1. All procedures followed were in accordance with the ethical standards of Wake Forest University. The study was approved by the institutional review board of Wake Forest University and all participants provided informed consent prior to participation in the study.

### 2.2. Study Design

This study was designed as a cross-over trial, with each participant receiving, in a randomized order, a single dose of Beet It Sport^®^ (James White Drinks Ltd.; Ipswich, United Kingdom) alone; Beet It Sport^®^ plus vitamin C supplement; and Beet It Sport^®^ plus a whey protein supplement. Each Beet It Sport contained 380 mg of nitrate.

### 2.3. Protocol

Participants reported to the Clinical Research Center of the Department of Health and Exercise Science at Wake Forest University on three separate occasions. Individuals were initially prescreened to determine eligibility. If eligible, they were instructed to report to a testing facility in an overnight fasted condition and to avoid high-nitrate-containing foods. At this first visit, informed consent was obtained. To determine eligibility, participants completed self-report questionnaires regarding health history and current health status, prescription and non-prescription medication use over the past 2 weeks, and recent dietary nitrate/nitrite intake for the past 24 h. A baseline blood draw was taken from the antecubital vein and stored in two 4 mL lithium heparin vials. Blood samples were immediately centrifuged at 1700× *g* for two min. Using a micropipette, two 0.4 mL aliquots of plasma were removed and stored in a -80 degree Celsius freezer. Plasma was thawed, treated with equal volume of methanol and centrifuged at 11,000× *g* for 10 min. Nitrate and nitrite concentrations were analyzed in supernatants by an ENO-20 nitric oxide analyzer (Amuza Inc. San Diego, CA, USA).

In randomized order, the participants then consumed 70 mL of Beet It Sport^®^ drink containing 380 mg of nitrate, either alone, in combination with a 500 mg tablet of vitamin C or in combination with 15 g of whey protein supplement mixed with 180 mL of water. Participants were requested to consume intervention supplements within 15 min. At 1 h and 3 h after consuming the randomized intervention treatment, blood was drawn again from each participant, and processed and stored as described above. There was a minimum of 2 days and a maximum of 1 month of washout between each of the 3 treatments. Plasma nitrate and nitrite levels were examined at two different time points, 1 and 3 h after ingestion of the intervention products. This is based on the pharmokinetics of nitrate and nitrite showing peak responses occurring during this time after ingestion of a nitrate-containing product [33]. Based on increases in plasma nitrite achieved using tolerable volumes of beetroot juice by other studies [12,47,48,49,50], participants who had a 3 h plasma nitrite level of 0.50 mM or greater were deemed as a “responder” and a plasma nitrite level of less than 0.50 mM at 3 h were deemed as a “non-responder”. The nitrate and nitrite micromolar concentration was quantified in both the pre-consumption and post-consumption blood draws by a ENO-20 nitric oxide analyzer, as described earlier. The intraclass correlation coefficients for the day-to-day test–retest reliability in our laboratory was 0.987 for nitrate and 0.987 for nitrite.

### 2.4. Analytic Plan

Plasma nitrate and nitrite at each time point for the three treatments were tested for normality using Shapiro–Wilk test. Since not all variables were normally distributed (*p* < 0.05), they were transformed by computing the log of the variable. The transformed data were tested for normality using Shapiro–Wilk, and all log-transformed variables were normally distributed (*p* < 0.05). Group means for log plasma nitrate and log plasma nitrite at pre- and 1 h and 3 h post-consumption were compared using two-way within-factors ANOVA, with time and treatment as the independent factors. Values for plasma nitrate and nitrite are presented in the text and table as unadjusted means ± SEM for ease of readers to understand values. Spearman correlations were performed between percent change in plasma nitrate and plasma nitrite, as not all of these variables were normally distributed as assessed using the Shapiro–Wilk test (*p* < 0.05). All analyses were performed using SPSS^®^ version 28.0 (Chicago, IL, USA), and statistical significance was set at *p* < 0.05.

## 3. Results

A total of 12 participants (10 women and 2 men) consented and completed all three interventions and data collection at each time point. The mean age was 67.6 ± 6.5 years.

Unadjusted treatment means for plasma nitrate and plasma nitrite at each time point for each intervention are shown in Table 2, while the statistical analysis was performed on the log transformed values. Using a Greenhouse–Geisser correction, no significant interaction was found between time (i.e., pre, 1 h and 3 h) and treatments (BRJ, BRJ + Vit C, BRJ + Protein for log plasma nitrate (*p* = 0.920) or for log plasma nitrite (*p* = 0.743)). There were no statistically significant differences for log plasma nitrate (*p* = 0.308) or log plasma nitrite (*p* = 0.391) values across treatments. Time means (Table 2) for log plasma nitrate increased significantly from pre-consumption levels after 1 h (*p* < 0.001). At 3 h, log plasma nitrate values had decreased significantly from 1 h values (*p* < 0.001) but were still significantly greater than pre-consumption values (*p* < 0.001). Log plasma nitrite values were significantly greater at 1 h (*p* = 0.001) and 3 h (*p* < 0.001) compared to pre-consumption levels. Log plasma nitrite values were significantly greater at 3 h compared to 1 h (*p* = 0.003).

Spearman correlations between percent change of plasma nitrate and nitrite from baseline values at 1 h and 3 h were examined to describe factors that influence plasma nitrate and nitrite responses. Since there were no differences between groups in the nitrate and nitrite responses, Pearson correlations were performed across all three interventions for percent change from pre- to 1 h and 3 h post-consumption in plasma nitrate and plasma nitrite. A greater percentage change in plasma nitrate at both 1 h and 3 h post-consumption was statistically significantly correlated with a greater percentage change in plasma nitrite at 3 h (r_s_ = 0.435; *p* = 0.008 for 1 h and r_s_ =0.461; *p* = 0.005 for 3 h), but not for plasma nitrite at 1 h.

**Figure 1:**
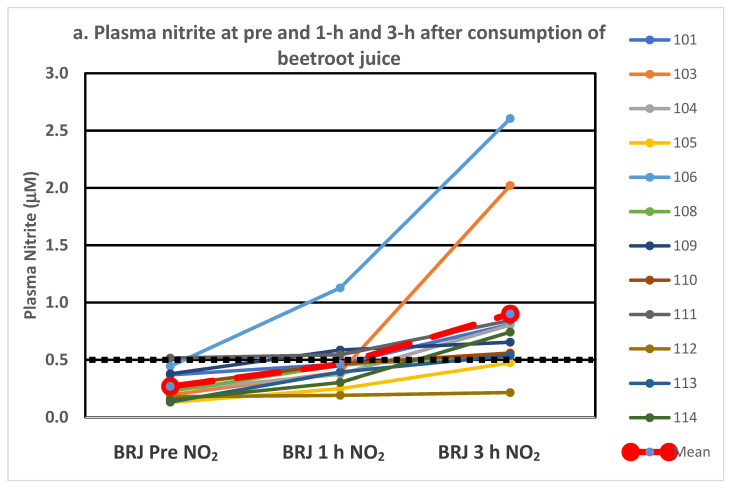

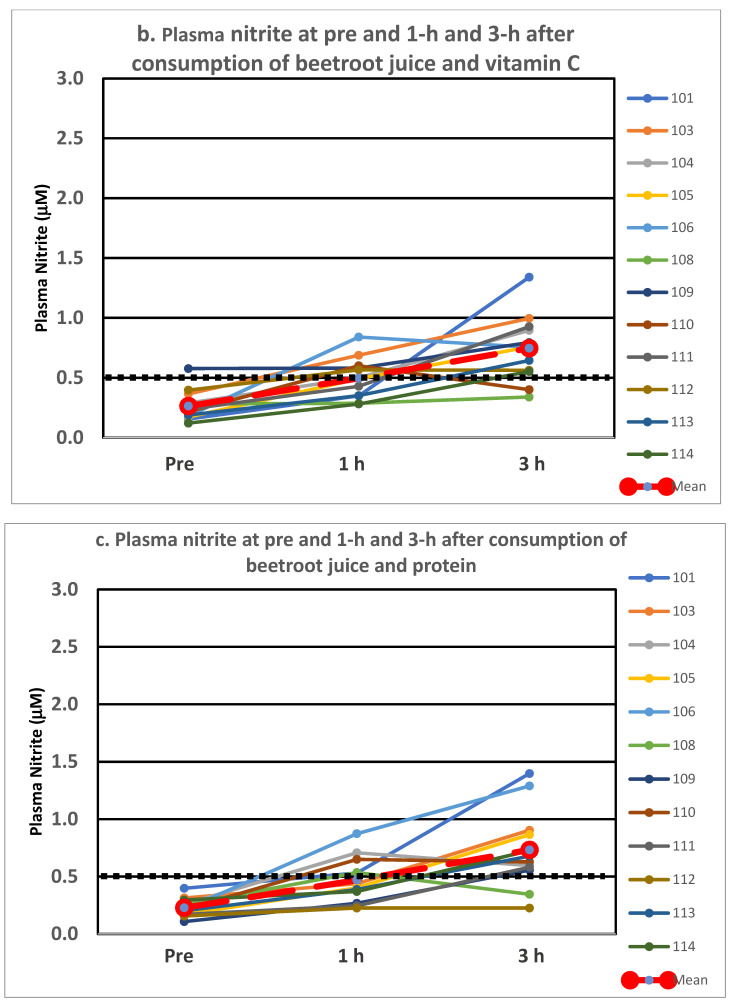
(**a**–**c**). Individual responses in plasma nitrite following consumption of beetroot juice alone (**a**), with vitamin C (**b**) and with protein (**c**). The treatment mean is depicted in the large dashed red line and the black dotted line at 0.5 mM line shows the level marking responders vs. non-responders.

Individual plasma nitrite levels at pre and at 1 h and 3 h post-consumption are depicted in Figure 1a–c for beetroot juice alone, with vitamin C and with protein, respectively. Two of the twelve participants within each treatment were deemed non-responders (3 h plasma nitrite level < 0.50 μM), with 10 classified as responders (3 h plasma nitrite level > 500 μM). Categorizing an individual as a responder or a non-responder was based on the definition employed in our study. Two separate participants failed to achieve the 0.50 μM level at 3 h for two of the treatments, and two others failed to achieve the 0.50 mM level on just one of the treatments. It is interesting to note the variability in the figures within and between individuals for the various treatments.

## 4. Discussion

This analysis compared the effect of beetroot juice alone or in combination with either vitamin C or protein in a randomized cross-over trial of healthy middle aged and older adults. These comparisons were based on the premise that: (1) vitamin C alters nitrite metabolism; and (2) both beetroot juice and protein are popular supplements for athletes, and are therefore likely to be consumed simultaneously. Our results suggest that in this cohort and conditions of the interventions and study design, neither vitamin C nor protein co-ingestion with beetroot juice alters plasma nitrate and nitrite response compared to beetroot juice alone.

Consistent with our findings, Ashor et al. found no differences in plasma nitrate and nitrite levels measured 3 h after consuming 0.1 mmol potassium nitrate/kg body weight alone or with a 200 mg dose of vitamin C in both younger and older adults [51]. Interestingly, they observed a synergistic physiologically effect with regard to improvements in arterial stiffness and blood pressure in older adults compared to vitamin C or nitrate alone. This suggests that although the biomarker for nitric oxide (plasma nitrite) is not changed with the co-ingestion of vitamin C and a dietary nitrate source, the physiological action of nitric oxide is enhanced with vitamin C consumption. Our study did not measure any physiological effects, such as blood pressure, from the treatments; therefore, it is not known if vitamin C plus beetroot juice in our study led to enhanced physiological actions. In contrast, others found greater change in fasting levels of plasma nitrate from baseline to 4 weeks of supplementation with co-administration of 1000 mg of vitamin C and a 6.4 mmol dose of nitrate in beetroot juice compared to nitrate alone [52]. Surprisingly, the change Basaqr et al. found in fasting plasma nitrite was greater for the nitrate supplement alone. It is interesting to note they report fasting levels for plasma nitrite 2–3-fold higher (0.4–0.5 mM) than values frequently found in the literature [12,26,49,53,54]. Furthermore, Basaqr et al. also showed that the combined levels of both nitrate and nitrite (NOx) in plasma correlated significantly with plasma vitamin C levels after the combination treatment [52]. In this group of hypercholesterolemic middle-to-older aged adults, the combined treatment of vitamin C and beetroot juice reduced blood lipids and oxidized low-density lipoproteins [52]. Together, this supports the epidemiological evidence that consumption of fruits and vegetables, which are high sources of vitamin C and nitrate, lowers the risk of cardiovascular disease [3,4].

Vitamin C’s action in the nitrite metabolism includes blunting the formation of N-nitroso compounds [46,55,56] through stimulating the conversion of nitrite into nitric oxide [44]. Nitrous acid forms dinitrogen trioxide (N_2_O_3_), which reacts with amines to form nitrosamines [33,46]. Vitamin C undergoes nitrosation in the presence of nitrous acid (HNO_2_) and forms nitric oxide. Thus, with vitamin C there is less nitrous acid and less nitrosamine formation. Reducing the formation of these carcinogenic nitrosamines is considered advantageous [44]. Increasing the nitric oxide pool with vitamin C and nitrate supplementation may explain the improvement in blood pressure and arterial stiffness previously observed [51].

Currently, research does not indicate that dietary protein affects the metabolism of nitrate in the nitrate–nitrite–nitric oxide pathway, such as reducing or increasing plasma levels of nitrate or nitrite. It is recognized that amines in amino acids react with N_2_O_3_ to form nitrosamines [33,46] in an acidic environment in the stomach, which in theory would reduce the level of plasma nitrite, since there would be less nitrite available for absorption. The current results do not support this reduction as plasma nitrite levels were similar among beetroot juice alone and in combination with a protein supplement.

As shown in Figure 1a–c, 2 of the 12 participants (~17%) in each treatment were classified as non-responders based on our definition employed in this study as they did not achieve a plasma nitrite level of at least 0.50 μM at the 3 h time-point. This frequency is similar to our earlier work, which showed approximately 20% of participants had suboptimal responses [53,57]. Several factors are known to affect the nitrate–nitrite–nitric oxide metabolism, including composition and abundance of oral and gut microbes, use of hexedine-containing mouthwash, spitting, saliva flow rate, smoking, source of nitrate, dietary components (vitamin C, antioxidants, polyphenols and thiocyanates), digestion of the food bound nitrate to free inorganic nitrate, and stomach pH [58,59,60,61,62]. Efforts were made to minimize several of these factors through inclusion/exclusion criteria (non-tobacco users, no active gastrointestinal disorders, not taking medications suspected to alter gut pH), instructions to avoid use of mouthwash and maintaining a similar diet for the different treatments. Furthermore, the treatment order was randomized, and a washout period was employed in the research design.

It is recognized that several individual biological factors and physical and chemical characteristics of the treatments (beetroot juice, vitamin C + beetroot juice, and protein + beetroot juice) potentially influence plasma nitrite levels. The composition of the oral microbes, use of antibacterial mouthwash, spitting, saliva flow rate, smoking, source of nitrate, dietary components that interact with nitrate metabolism (vitamin C, antioxidants, polyphenols, and thiocyanates), digestion of the food bound nitrate to free inorganic nitrate, gut microbiome composition and abundance, stomach pH [33,59,60,61,62,63,64,65]. Gastric emptying rate is also a key factor, especially in a time course study such as this. The speed at which the stomach empties its content into the duodenum is influenced by several factors, including the fluid volume, osmolality of the food bolus and age [66,67,68]. While the inclusion and exclusion criteria and study design were developed to minimize several of these factors, attempts were not made to equalize the treatments based on differences in fluid volume and osmolarity, which may have influenced the results. Additionally, age is also a likely contributing factor to the plasma nitrate and nitrite response to nitrate consumption [36,38,69,70]. In the wide age spread of this cohort (40–80 years old), this also may have implications in interpreting the results.

## 5. Conclusions

Thus, considering the study design and interventions delivered, these results demonstrate in this cohort of middle-to-older aged healthy adults, the co-ingestion of beetroot juice with a 200 mg dose of vitamin C or 15 g of a protein supplement does not alter the efficacy of converting dietary nitrate to plasma nitrite within the 1 and 3 h sampling employed. Further research needs to be undertaken to expand the blood-sampling time-frame and to examine factors that may influence the kinetics of the plasma nitrate to nitrite efficacy, such as differences in fluid volume and osmolarity between treatments employed.

## Figures and Tables

**Table 1:** Inclusion and Exclusion Criteria.

Inclusion
Middle and older age (40–80 year-old) men and women Body mass index between 18.5–30.0 kg/m^2^ Able to provide own transportation to study testing visits Able to consume study beverages Able to speak and read English
**Exclusion**
Tobacco user (smoke or chew), including e-cigarettes Known conditions: diabetes mellitus (type 1 or 2); atrophic gastritis; hypo- or hyperthyroidism; gout; history of kidney stones; history of hypotension; cardiovascular disease; chronic obstructive pulmonary disease; inflammatory bowel diseases; impaired liver or kidney function Current or recent (last 3 months) treatment for cancer Current use of the following medications: Phosphodiesterase type 5 inhibitors; nitroglycerin or nitrate preparations; proton pump inhibitors; medication for hypothyroidism; antacid and heartburn medications

**Table 2:** Non-transformed intervention means for plasma nitrate and nitrite at pre- and 1 h and 3 h post-consumption of the intervention. Values for pre, 1 h post and 3 h post are mean ± standard error of the mean. The Time Means and Treatment Means are estimated marginal means ± standard error of the mean. All analysis were performed on log-transformed values for plasma nitrate and nitrite, but the non-transformed values are presented for ease of reader interpretation. Values with different superscript letters within a column were statistically significantly different from each other.

	Beetroot Juice	Beetroot Juice + Protein	Beetroot Juice + Vitamin C	Time Means
Plasma Nitrate (μM)				
Pre	52.3 ± 12.8 ^a^	63.0 ± 16.7 ^a^	43.0 ± 5.8 ^a^	52.8 ± 7.9 ^a^
1 h Post	657.2 ± 44.6 ^c^	696.5 ± 45.6 ^c^	602.7 ± 42.2 ^c^	652.1 ± 33.0 ^c^
3 h Post	508.2 ± 39.0 ^b^	558.4 ± 38.7 ^b^	504.2 ± 29.0 ^b^	523.6 ± 30.1 ^b^
Treatment Means	405.9 ± 30.5	439.3 ± 29.7	383.3 ± 22.6	
Plasma Nitrite (μM)				
Pre	0.270 ± 0.037 ^a^	0.229 ± 0.023 ^a^	0.266 ± 0.037 ^a^	0.255 ± 0.017 ^a^
1 h Post	0.462 ± 0.069 ^b^	0.470 ± 0.057 ^b^	0.497 ± 0.049 ^b^	0.476 ± 0.048 ^b^
3 h Post	0.901 ± 0.200 ^c^	0.733 ± 0.099 ^c^	0.747 ± 0.080 ^c^	0.794 ± 0.106 ^c^
Treatment Means	0.555 ± 0.092	0.477 ± 0.050	0.503 ± 0.050	

## Data Availability

The data presented in this study are available on request from the corresponding author.
